# Evaluation and construction of the capacities of urban innovation chains based on efficiency improvement

**DOI:** 10.1371/journal.pone.0274092

**Published:** 2022-10-26

**Authors:** Rui Zhang, Changxu Ji, Liguo Tan, Yuqin Sun

**Affiliations:** 1 College of Tourism & Landscape Architecture, Guilin University of Technology, Guilin, Guangxi, China; 2 Guangxi Tourism Industry Research Institute, Guilin, Guangxi, China; Shenzhen University, CHINA

## Abstract

This study examines urban innovation activities from the perspective of improving the efficiency of innovation chains and explored their operational patterns. The connotation of the urban innovation chain was analyzed following the process of the innovation chain and the characteristics of urban innovation activities. The urban innovation chain can be divided into the following three stages: original, technological, and innovation transformation. An indicator system for evaluating the three capacities of the urban innovation chain was established. Furthermore, the development level of strategic emerging industries was used as an indicator to measure the chain’s efficiency and analyze the relationship between these three constructed capacities in Chinese national innovative cities. Data published on the national innovative cities were used to measure the capacity of the urban innovation chain. The development of Strategic emerging industries indicates cities’ innovation performance, and an inductive approach was applied to analyze the relationship between the capacity construction of the urban innovation chain and innovation performance in innovative cities. This study found that the capacity of the urban innovation chain positively affected urban innovation performance and that the balanced development of the three capacities positively affected urban innovation performance. When urban innovation performance was at its highest, the contribution of original innovation capacity to innovation performance was significantly greater than that of the other two capacities; thus, an inverted U-curve illustrates the relationship between the improvement of urban innovation performance and the balance of the three capacities of the urban innovation chain. This study’s conclusions enrich the relevant theories of the innovation chain, provide a reference for the construction of the urban innovation chain, and promote the development of Strategic emerging industries.

## Introduction

Cities play an important role in competition for regional innovation capacities. Once a city has achieved remarkable results in global competition, it can attract more high-quality innovation resources, which will not only further promote the city’s innovative development, but also provide positive feedback to other cities as a result of the spillover effect, enhancing their regional innovation capacity [1]. In 2008, China started to build national innovative cities. Currently, 78 national innovative cities have concentrated 77.2% of their national R&D expenditure, held over 85% of China’s valid patents, and nurtured more than 80% of China’s high-tech enterprises, demonstrating remarkable innovation performance [2]. At present, national innovative cities have become important clusters of innovation resources in China; however, due to differences in scientific, educational, and industrial bases, each innovative city has formed a unique pattern for innovative development with different innovative functions.

Strategic emerging industries (SEIs) are those that achieve intensive integration of emerging technologies and industries [3]. They are important forces that guide future economic and social development, and they constitute important elements in the construction of innovative cities in China. In the construction of the regional innovation system, innovation activities and various innovation agents are not fragmented but are closely connected in the form of a chain, which is known as the innovation chain [4]. National innovative cities have formed urban innovation networks to support regional economic development in China [5]. Innovation activities and agents in cities are involved in the innovation chain of the regional innovation system, and there are multiple innovation chains (or numerous links in the innovation chain network) in one city. The progress of digital information technology has transformed and developed SEIs [6].

Existing research has recognized the importance of SEIs in the construction of innovative cities in China. Technological innovation, industrial structure upgrading, and economic development mode transformation brought about by the development of SEIs can enhance a city’s independent innovation capacity [7]. This reveals that SEIs and innovative cities are symbiotic co-evolutionary systems being in a relationship of mutual coupling and promotion [8]. The evolutionary power of its coupling system comes mainly from industrial upgrading, independent innovation, technological innovation, and environmental innovation [9]. The coupling pattern is typically expressed as a distribution state of points, lines, axes, bands, and areas [10]. Meanwhile, as SEIs are knowledge- and technology-intensive industries oriented toward technological innovation, the general consensus is that the processes of technological breakthroughs, R&D innovation, innovation transformation, and product innovation are presented in the form of innovation chains [11–13]. Scholars have explored the functional structure [14, 15], evolutionary laws and patterns [16, 17], operations and efficiency [18–20], and industrial innovation networks [21, 22] of the innovation chain of SEIs. However, existing research lacks the exploration of the construction, operational efficiency, and evolution process of the urban innovation chain (UIC) according to the characteristics of regional innovation activities. Different types of SEI innovation clusters in cities participate in innovation activities at various stages of the innovation chain. Urban innovation capacity is essentially the collection of the innovation capabilities of various organizations in the city.

**Fig 1:**
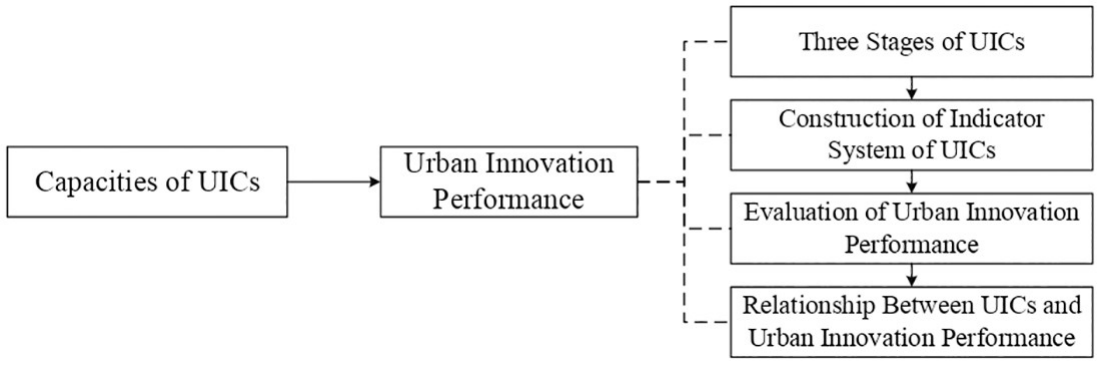
Overall research idea.

What are the essential properties of UICs? What strategy is used to construct the operational capacity of UICs? How can the operational efficiency of UICs be improved? To answer the above questions, this study analyzed the connotation, process stages, and evaluation indicators of UICs according to the characteristics of urban innovation activities, and examined the relationship between the construction and efficiency of UICs in innovative cities. This research will help optimize the layout of UICs in cities and promote the development of SEIs. Fig 1 illustrates the research idea. The remainder of this paper is organized as follows. Section 2 reviews the literature. Section 3 proposes the theoretical analysis and hypotheses. Section 4 presents an indicator system for the evaluation of UICs, indicators for evaluating urban innovation performance, and the measurement of the three capacities of UICs. Section 5 presents the results and hypothesis testing. Section 6 discusses the study implications and limitations.

## Literature review

The construction of innovation chains aims to improve the capacity for technological innovation and optimize innovation systems. From the perspective of enterprises, Sen (2003) [23] proposed that the innovation chain is a model for organizing innovation activities, which is a value-added process of innovation with market demand as the guide, technological innovation as the basis, the improvement of the competitiveness of enterprises as the goal, the participation of multiple agents at different stages and the continuous evolution of functional nodes. From the knowledge innovation perspective, Cai (2002) [24] points out that the innovation chain is a structural model of a functional chain, which takes a certain innovation subject as a core and connects related innovation subjects through knowledge innovation to realize the process of the knowledge economy and the optimization goal of the innovation system. Li (2018) [25] defined the innovation chain from a process perspective as the process from basic scientific research to the formation of scientific knowledge, which guides technological innovation and ultimately leads to mass market applications.

In summary, the innovation chain mainly reflects the flow, transformation, and value realization processes of knowledge and technologies in the process of commercialization. Its core is the cooperation and connection of innovation agents at different links in the process of commercialization, and its essence is the coordinated R&D among innovation agents. In essence, the core concept of the innovation chain lies in the openness of innovation elements, synergy of overall operation, and value addition. In the innovation chain, all innovation agents are closely connected, and each innovation activity has upstream and downstream connections [26]. Likewise, all innovation achievements are products of multiple innovation resources acting together [27].

Most scholars are interested in the division of stages in researching the process characteristics of the innovation chain. Timmers (1999) [28] divided the innovation chain into three stages: basic R&D, technological development, and application. Hansen and Birkinshaw (2007) [29] and Wu and Lin (2014) [30] hold that the innovation chain consists of three stages: stimulation of creative ideas, transformation, and diffusion. Yu and Liu (2013) [31], on the other hand, divided the innovation process into three stages from the perspective of the innovation value chain: knowledge innovation, research innovation, and product innovation. The three-stage division method lays the foundation for the formation of a basic idea of the innovation chain.

To describe the process of the innovation chain in detail, scholars have proposed different ways of dividing the innovation chain into stages, such as the four-stage and five-stage methods, where stages are more finely divided and their functions are more clearly defined. Turkenburg (2002) [32] divided the innovation chain into four stages: research, development, diffusion, and application. Li, Deng and Xu (2015) [33] considered the innovation chain as a complete chain consisting of four links: the development of creative ideas, the materialization of knowledge, the conceptualization of products, and marketing, in which the innovation elements are combined to accomplish the innovation goal. Bamfield (2006) [34] divided the innovation chain into five stages from the perspective of business development: exploratory research, technological development, product pilot production, market launch, and production and sales. Lu and Qin (2017) [35] referred to the viewpoint of the innovation value chain and summarize the innovation chain into five stages: basic research, applied research, product development and pilot production, commercialization, and industrialization.

Although scholars have proposed more detailed ways of dividing the stages of the innovation chain, they continue to take a certain technology, a certain product, or an innovation agent as the subject of the research; they follow the basic progressive logic of the innovation chain that starts with basic research, goes through multiple links and multiple agents, and ends with commercialization.

## Theoretical analysis and research hypothesis

### The construction of UICs

From a macro perspective, innovation chains connect knowledge, innovation, wealth, economic development, and national prosperity [36]. From a micro perspective, because of the complexity of innovation activities and the multiplicity of innovation chain agents, the innovation chain is not a single linear chain connecting universities, research institutes, enterprises, and users, but it rather consists of social organizations such as the government, universities, research institutes, enterprises, intermediaries (e.g., the financial technology sector), trade associations, and consulting companies. It involves basic research, applied research, design and development, pilot production and improvement, production and sales, industrialization, and diffusion, presenting an open and complex net-like chain [37].

The specificity of UICs lies in the fact that, in terms of geographical scope, on the one hand, the innovation chain around a product, technology, or agent is not limited to one specific city; on the other hand, within the space of a specific city, there are multiple nodes of innovation chains at different stages. To improve cities’ innovation capacities, they need to continuously form new, efficiently functioning innovation chains in the city or between the city and its outside. UICs are characterized by the continuous evolution and smooth connection of multiple innovation chains, the constant emergence of basic research innovations, and the steady formation of new products and industries, forging a platform for the full integration of innovation, industrial, and financial chains.

Therefore, this study analyzed the concept of UICs. In a narrow sense, UIC refers to the innovation chain of basic scientific research, technological innovation, and eventually large-scale industrial applications formed within the city. The global dissemination and diffusion of knowledge and international co-operation within scientific research have integrated regional innovation activities into the global innovation chain [38], and advances in digital technologies have enhanced the mobility and availability of innovation resources. Innovation chains are not limited to a particular city, but exist across cities and regions [39]. Therefore, this study considers UICs in a broad sense to mean that basic scientific research, technological innovation, industrialization, and application activities in cities are embedded in cross-regional innovation chains.

### Formulation of research hypotheses

Fundamentally, the core of the innovation chain model lies in the openness of innovation elements, synergy of the overall operation, and value addition [40]. When a city and an external region jointly build an innovation chain centered around a new technology, a new product and an innovative agent, governments at all city levels, various universities and research institutes, and various enterprises participate in the operation of the innovation chain. The construction of UICs provides opportunities in the urban space to transform original innovation into technological innovation, and to connect new technologies with enterprises’ demands. The innovation platform provides a space for fair competition between new technologies and existing technologies, and successful new technologies will be retained and applied, while unsuccessful ones will be disused. The government supports strategic emerging technologies with the potential to promote economic development through policies so that the innovation platform generates market demand with a scale conducive to commercialization. Therefore, the construction of UIC first requires an external environment to provide a good coordination mechanism and information-sharing platform to create conditions for new knowledge, technologies, and products. Second, the construction of all links in the innovation chain needs attention so that the city has the capacity to connect the innovation chain at all stages. Third, sufficient resources must be provided to ensure the smooth commercialization of new technologies through the innovation chain. The clustering of innovation resources with the city as the unit requires partnerships between the government, research institutions, industries, and intermediaries. Base on openness and synergy, each link in the innovation chain generates value and ultimately enhances overall value [41].

**Fig 2:**
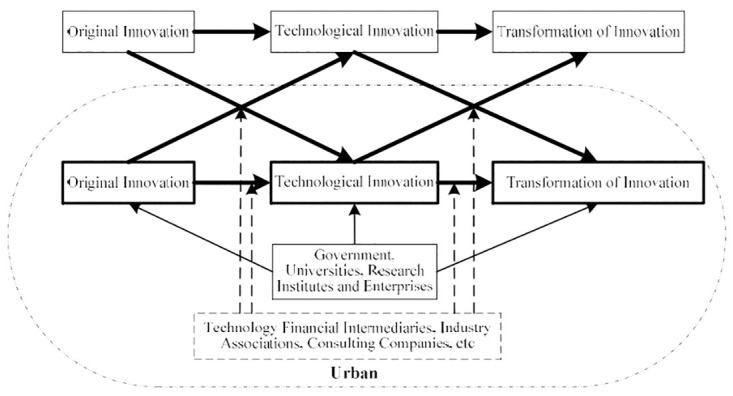
Construction of the UIC.

Innovation is one of the driving forces of sustainable regional development. While focusing on general innovation activities, the study of regional innovation capacity should focus on the three main links of science, technology, and industry from the perspective of the innovation chain [42]. The stage of original innovation in the UIC entails basic research and its results cannot be quantified in the short term; however, it has a long-term impact on the regional economy, social progress, and people’s lives. Putting it into the UIC system meets the interests of the cities. The stage of technological innovation revolves around applied research, which is the basis for the creation of new products and technologies and has an important impact on industrial application at later stages of the innovation chain [43]. The transformation of new technologies is a technical activity to transfer research results to products, and national and local governments have introduced many policies to promote the transformation of scientific and technological innovations. In summary, this study proposes that UIC can be divided into three stages: original, technological, and transformation of innovation (see Fig 2).

Innovation chains are fluid, loose innovation networks involving multiple innovation agents, which cooperate on the basis of sharing resources and advantages, and they are also free to choose to participate and withdraw. Innovation is a synergistic system between R&D and commercialization [44]. There are individuals and groups with different development goals in a region, so it is a complex problem to mobilize the innovation motivation of each type of agent to conform to the overall development goals [45]. The purpose of constructing innovation chains is to achieve a high degree of sharing of resources and advantages, such as knowledge, among innovation agents through cooperation and coordination to shorten innovation cycles, reduce innovation costs, and enhance innovation capacities and performance. Therefore, to realize the orderly connection of the three stages of the innovation chain and form an urban innovation platform with efficient operation, the improving the original, technological, and innovation transformation capacity is extremely important.

The UIC proposal provides an analytical framework for the study of urban innovation performance, which shows the complex relationship between the innovation capacity of each link in the innovation chain, the overall operational capacity of the chain, and innovation performance. To evaluate the efficiency of UICs, it is necessary to consider the level of development of the three capacities of UICs, analyze the impact of the operational capacity of UICs on innovation performance, and then build a new urban innovation structure to realize an effective connection between UICs and internal and external, as well as upstream and downstream, innovation resources.

Universities and research institutes have a large number of innovative talents and advanced research equipment, and master cutting-edge core technologies and theoretical knowledge, and they provide intellectual assistance for innovation. Enterprises have sufficient R&D funds, complete production and testing equipment, and rich marketing experience, providing market guidance for innovation. The government reduces information search costs among innovation agents by formulating relevant policies and regulations and building collaborative innovation platforms, thereby facilitating innovation activities [46, 47]. A complete innovation chain is an open and complex net-like chain that transcends city boundaries. There are multiple links and agents in the cities at different stages of the innovation chain. Among them, basic research can effectively promote technological innovation [48], the diffusion of technological innovation promotes the formation and development of new industries [49], and the stage of industrialization of new technologies reflects the operational efficiency of each innovation chain. Therefore, each innovation agent has different functions in the three stages of the UIC, and they influence each other and jointly complete the process from basic research to industrial application. An efficient UIC should build a ‘bridge’ among original innovation, technological innovation, and innovation transformation, promoting the creation of new knowledge by basic research institutions, guiding market-oriented technological innovation activities by industry and academia, and promoting the application and transformation of innovation results. Therefore, this study proposes the following hypothesis:

**H1:** The capacity of the UIC will positively influence urban innovation performance.
**H2:** Balanced development of the three capacities of the UIC will positively affect urban innovation performance.

Original innovation is the source of scientific and technological progress and constitutes the key to achieving innovation-driven economic development by providing advanced theoretical support for the development of science and technology [50]. Industrialized countries place more emphasis on basic research and the creation of new knowledge because basic research can provide a long-term knowledge base for enterprises’ technological innovation [51]. Studies have found that countries that have modernized through scientific and technological revolutions have strong scientific bases. More than 50 percent of the economic growth of the United States in the last 25 years can be attributed to R&D driven by basic research [52]. Original innovation capacity determines the level of regional economic and social development. Therefore, we propose the following hypotheses:

**H3:** The higher the capacity of the UIC, the greater the contribution of the original innovation capacity to innovation performance.

## Method

### An indicator system for the evaluation of UICs

Evaluating the capacity and operational efficiency of each stage of the UIC can help clarify the urban innovation structure, construct an urban technology policy system, and improve the efficiency of the UIC. After reviewing the relevant evaluation systems for urban innovation capacity, we found that the evaluation indicators generally involved innovation resources input [53, 54], innovation results output [55–58], innovation environment [59–62], and innovation enterprises [63, 64]. This study established an indicator system for evaluating UIC by combining urban innovation practices with relevant literature. Considering the authority, influence, and timeliness of the indicators, authoritative innovative evaluation indicators, such as the Global Innovation Hubs Index 2020 (CIDEG) [65], Global Innovation Index 2020 (WIPO) [66], Innovation Cities Index 2020 (2thinknow) [67], and the research results of related scholars were analyzed. The indicators related to the original urban innovation capability, technological innovation capability and innovation transformation capacity were extracted for reference, and 14 secondary indicators were determined (see Table 1).

**Table 1:** Evaluation index system of UICs.

No.	Primary Index	Secondary Index	Literature Sources
1	Original Innovation Capability	The Ratio of R&D Expenditure to Regional GDP	Fang, Ma, Wang and Li (2014) [68]; Wan, Liu and Gu (2016) [69]; Liu, Yang and Xiao (2021) [70]
		The Proportion of Basic Research Expenditure to R&D Expenditure	Xu and Zhou (2008) [71]; Liu and He (2011) [72]; Wang (2011) [73]; Jiang and Zha (2016) [74]
		The Ratio of R&D Personnel Among 10,000 Employed Personnel	Fang, Ma, Wang and Li (2014) [68]
		Number of National Science and Technology Awards	Zhang (2010) [75]; Cai, Liu and Huang (2021) [76]
2	Technological Innovation Capability	The Ratio of R&D Expenditure to The Main Business Income of Industrial Enterprises Above the Designated Size	Li and Fu (2015) [77]; Lin, Zhang and He (2020) [78]
		The Number of High-tech Enterprises	Felsenstein (2015) [79]; Yang and Li (2020) [80]
		The Ratio of Business Income of National High-tech Zones to Regional GDP	Fang, Ma, Wang and Li (2014) [81]; Xiao, et al. (2021) [82]
		The Number of Patents Owned By 10,000 People	Xu, Xu and Wu (2014) [83]; Fang, Ma, Wang and Li (2014) [68]; Qiu, et al. (2020) [84]; Zheng and Wu (2021) [85]
		The Ratio of Technology Output Contract Turnover to Regional GDP	Hou, Liu and Zhang (2017) [86]; Zheng and Wu (2021) [85]
3	Innovation Transformation Capacity	The Ratio of Technology Input Contract Turnover to Regional GDP	Wang, Zhang and Zhang (2021) [87]
		The Number of Listed Companies Under the Science and Technology Sector	Zhang (2010) [75]
		The Number of National Science and Technology Business Incubators, Science and Technology Parks Affiliated to Universities and Demonstration Bases for Entrepreneurship and Innovation	Fang, Ma, Wang and Li (2014) [81]; Tsai and Wang (2005) [88]
		The Number of Science and Technology-based SMEs	Yang and Li (2020) [80]
		The Ratio of New Product Sales Revenue to Main Business Revenue of Industrial Enterprises Above Designated Size	Yu and Liu (2013) [89]; Wang, et al. (2016) [90]; Felsenstein (2015) [79]; Zhou and Li (2011) [91]; Sun, et al. (2012) [92]

Original innovation capacity is measured by the input and output of scientific and technological resources. Given the different sizes and levels of cities [60], we mainly used relative indicators and relevant indicators of local governments and enterprises as the main agents of innovation [45]. The ratio of R&D expenditure to regional GDP reflects the degree of importance that society attaches to R&D, avoiding errors caused by different sizes of cities [69]. The proportion of basic research expenditure to R&D expenditure reflects the intensity of investment in the original innovation [73, 74]. The ratio of science and technology personnel to the employed population measures the proportion of the input of human resources in science and technology in the region. The higher the ratio, the more R&D personnel the city has and the stronger its innovation capacity. Therefore, three indicators for the input of scientific and technological resources were set: the ratio of R&D expenditure to regional GDP, the proportion of basic research expenditure to R&D expenditure, and the ratio of R&D personnel among 10,000 employed personnel. To reflect the city’s output of high-quality original innovation, the output of scientific and technological resources was measured by the “number of national science and technology awards” the city had received.

Technological innovation capacity is measured by a city’s technological innovation inputs and outputs. The ratio of R&D expenditure to the main business income of industrial enterprises above a designated size reflects the importance that enterprises attach to R&D and the intensity of innovation activities inside and outside these enterprises. The number of high-tech enterprises reflects the scale and environment of innovative enterprises in cities, which are important carriers of applied R&D. Therefore, the ratio of R&D expenditure to the main business income of industrial enterprises above the designated size and the number of high-tech enterprises are indicators of technological innovation input. The ratio of the business income of national high-tech zones to regional GDP was used to measure the economic efficiency of innovative industries in cites [82]. The number of patents owned by 10,000 people indicates technological innovation capacity and efficiency [84]. The ratio of technology output contract turnover to regional GDP reflects the value of technology output and the capacity of technology output in the market [86]. The ratio of annual regional technology output contract turnover to regional GDP measures both the commercial value of technology output in different regions and the capacity of the technology output to the market [93]. Therefore, technological innovation output is measured by “the ratio of business income of national high-tech zones to regional GDP,” “the number of patents owned by 10,000 people,” and “the ratio of technology output contract turnover to regional GDP.”

The turnover of technology input contracts reflects the firm’s ability to import technological achievements. Science and technology parks promote cooperation among industries, universities, and high-tech enterprises [88]. The income from selling new products reflects the economic contribution of the industrialization of technological innovations [91, 92]. Therefore, the “ratio of technology input contract turnover to regional GDP,” “the number of listed companies under the science and technology sector,” “the number of national science and technology business incubators, science and technology parks affiliated to universities and demonstration bases for entrepreneurship and innovation,” the “number of science and technology-based SMEs (small and medium-sized enterprises),” and the “ratio of new product sales revenue to main business revenue of industrial enterprises above designated size.”

### Indicators for evaluating urban innovation performance

SEIs play a leading role in the overall and long-term development of national economies and societies. After 10 years of development, China’s SEIs have become a powerful support for China’s industrial transformation and coordinated regional development. Innovation is the key to the development of SEIs. Therefore, this study uses the level of development of SEIs in cities as an index for evaluating urban innovation performance, and analyzes the relationship between the construction of the three capacities of the UIC and the development of SEIs. Among SEIs, high-end digital equipment in the manufacturing and digital economy industries has a significant impact on the improvement of comprehensive national power. Therefore, this study analyzes the industries of industrial robotics, integrated circuits, marine engineering equipment, high-end CNC machine tools, environmental protection equipment, and the digital economy, which immensely drive economic growth and have a high industrial concentration. This study selects 115 leading manufacturers with advanced technology and significant influence in their industries and measures the innovation performance of cities by their distribution in these innovative cities.

### Measurement of the three capacities of UICs

According to the distribution of 115 leading manufacturers in SEIs, 21 national innovative cities with two or more leading manufacturers are studied here, and the data for constructing the indicators of the three capacities of UICs are retrieved based on the 2020 Report on the Innovation Capacity of National Innovative Cities (see Table 2).

**Table 2:** Three capabilities data of UICs and the number of leading manufacturers in SEIs.

No.	City	The Number of Leading Manufacturers in SEIs	Original Innovation Capacity	Technological Innovation Capacity	Innovation Transformation Capacity
1	Beijing	18	280.7	210.42	127.39
2	Shanghai	16	187.72	126.62	97.06
3	Shenzhen	10	77.23	84.57	87.41
4	Hangzhou	7	73.12	71.15	80.29
5	Nanjing	5	83.43	72.68	74.73
6	Shenyang	5	67.49	59.15	64.7
7	Guangzhou	4	80.94	72.67	79.19
8	Dalian	4	65.21	62.63	48.45
9	Tianjin	3	72.17	63.64	51.08
10	Chengdu	3	71	66.1	71.58
11	Qingdao	3	71	65.47	68.12
12	Jinan	3	66.18	69.16	64.67
13	Yangzhou	3	42.51	56.1	46.28
14	Suzhou	3	54.15	68.72	82.25
15	Chongqing	2	90.21	18.52	36.75
16	Wuhan	2	77.52	70.39	74.93
17	Hefei	2	70.66	68.11	57.75
18	Kunming	2	61.8	56.48	50.38
19	Wuxi	2	59.44	65.57	67.01
20	Nantong	2	42.31	55.11	54.71
21	Baoji	2	25.47	39.8	21.89

Source: Industrial innovation and competition map (2018)

The 2020 Report on the Innovation Capacity of National Innovative Cities (2021).

**Fig 3:**
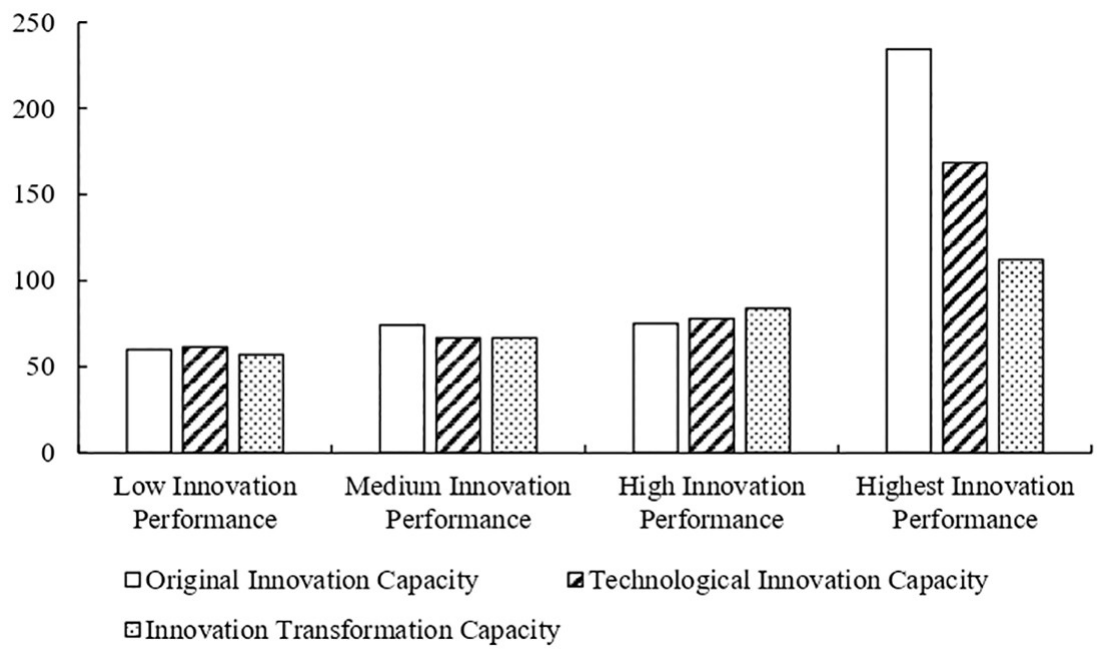
Three capabilities mean of UICs at different levels of innovation performance. Source: The authors.

The innovation performance of cities is measured by the number of leading manufacturers in SEIs; that is, cities with two to three leading manufacturers have low innovation performance, cities with four to five leading manufacturers have medium innovation performance, cities with seven to 10 leading manufacturers have high innovation performance, and cities with more than 10 leading manufacturers have the highest innovation performance. The averages of the three capacities of the innovation chain in cities with low innovation performance were 61.88, 58.71, and 57.49 respectively; 74.27, 66.78, and 66.77, respectively, in cities with medium innovation performance; 75.18, 77.86, and 83.85, respectively, in cities with high innovation performance; and 234.21, 168.52, and 112.23, respectively, in cities with the highest innovation performance (see Fig 3).

## Results

To test the research hypotheses, this study explored the relationship between the construction of the three capacities of UICs and the operational efficiency of innovation chains based on the distribution and quantitative indicators of leading manufacturers in SEIs in national innovation cities.

### Analysis of characteristics of UICs in cities with low innovation performance

**Fig 4:**
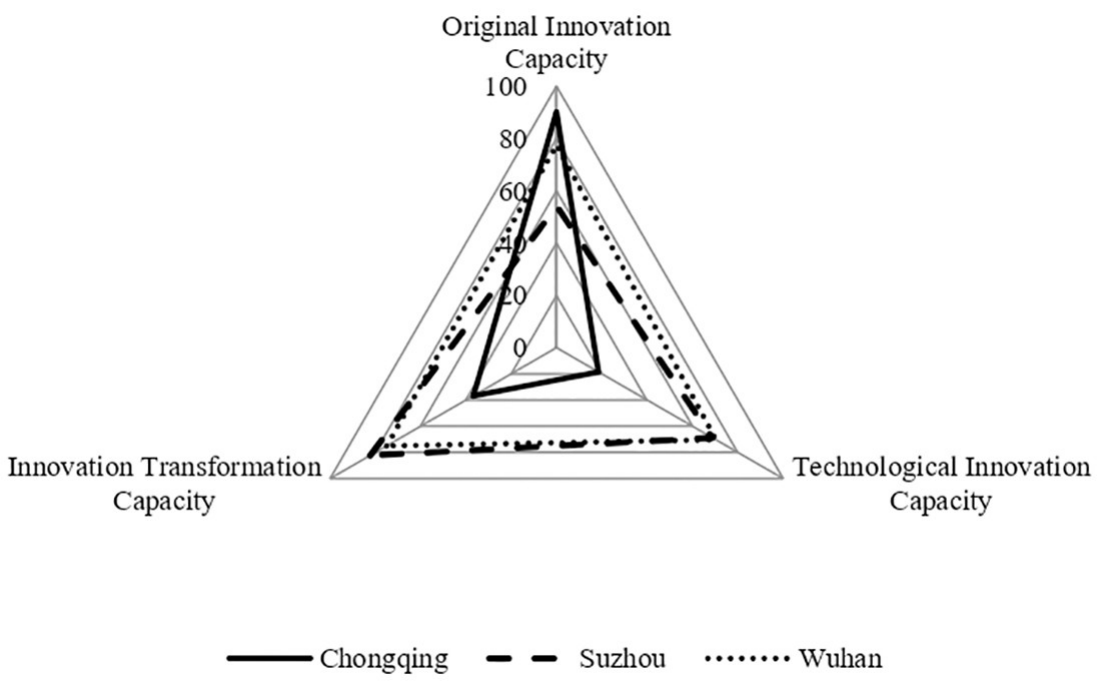
Three capacities of UICs in cities with low innovation performance.

Among the 13 cities with low innovation performance, 10 cities (Jinan, Chengdu, Qingdao, etc.) show low levels of original innovation capacity, technological innovation capacity, and innovation transformation capacity of the UIC. The averages of the three capacities were 58.25, 60.55, and 55.35, as shown in Table 2. Chongqing and Suzhou have uneven development of the three capacities, with Suzhou having outstanding innovation transformation capacity and Chongqing having a high original innovation capacity. Specifically, Suzhou’s innovation transformation capacity was 82.25, original innovation capacity was 54.15, and technological innovation capacity was 68.72; Chongqing’s original innovation capacity was 90.21, technological innovation capacity was 18.52, and innovation transformation capacity was 36.75. Wuhan had relatively high levels of all three capacities. Wuhan’s original innovation capacity was 77.52, technological innovation capacity was 70.39, and innovation transformation capacity was 74.93, ranking 6th, 7th and 7th, respectively, in terms of the three capacities of the UIC among the 21 national innovation cities. Wuhan has only two leading manufacturers of high-end CNC machine tools. The main reason is that Wuhan’s industrial layout is designed to establish industrial clusters such as optoelectronic information, automobiles and parts, and biomedicine, and to consolidate and upgrade traditional industries with advantages such as equipment manufacturing, steel and deep processing, food and tobacco, energy, home appliances, petrochemicals, textiles, clothing, and building materials. Wuhan only started to commit to the breakthrough development of its digital economy industry such as artificial intelligence, blockchain, cloud computing, big data, 5G, and so forth in 2020. Therefore, although the three capacities of Wuhan’s UIC are all at a high level, the low level of development of SEIs is affected by its urban industrial layout. The capacities of Chongqing, Suzhou and Wuhan are shown in Fig 4.

The analysis above shows that SEIs are at a relatively low level of development when either the three capabilities of the UIC are at a low level, one of the three capabilities is high and the other two capabilities are low, or the city lacks the layout of the digital economy industry. A single enhancement of original innovation or innovation transformation cannot promote the development of SEIs in cities. In summary, when the three capacities of the UIC are generally low or their development is uneven, the UIC operates less efficiently. The level of development of SEIs is influenced by the industrial layout of cities.

### Analysis of characteristics of UICs in cities with medium innovation performance

**Fig 5:**
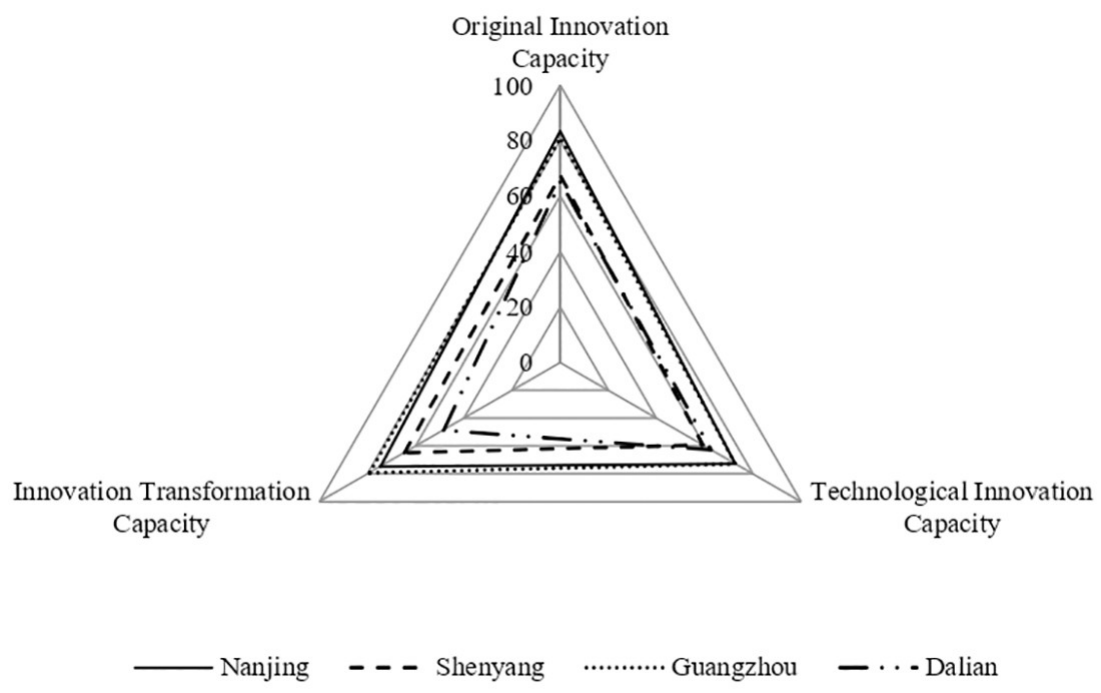
Three capacities of UICs in cities with medium innovation performance.

Nanjing, Guangzhou, Shenyang and Dalian have innovation performance, as shown in Fig 5. Nanjing’s original innovation capacity was 83.43, technological innovation capacity was 72.68, and innovation transformation capacity was 74.73, ranking 4th, 4th and 8th in terms of the three capacities of UICs among the 21 national innovation cities. Guangzhou’s original innovation capacity was 80.94, technological innovation capacity was 72.67, and achievement transformation capacity was 79.19, ranking 5th, 5th and 6th. The three capacities of the UICs in Nanjing and Guangzhou were relatively high and had a balanced development in each stage, and the operational efficiency of the UICs was at a medium level.

The three capacities of the UICs in Shenyang and Dalian were low. Shenyang’s original innovation capacity was 67.49, technological innovation capacity was 59.15, and innovation transformation capacity was 64.7, ranking 13th, 16th and 12th in terms of the three capacities of UICs among the 21 national innovation cities. Dalian’s original innovation capacity was 65.21, technological innovation capacity was 62.63, and innovation transformation capacity was 48.45, ranking 15th, 15th and 18th, respectively. These two cities do not have leading manufacturers in the digital economy industry. In terms of the characteristics of leading manufacturers in the digital high-end equipment industry, enterprises such as Siasun Robotics and Automation and Kingsemi in Shenyang were affiliated with the Chinese Academy of Sciences and were state-supported enterprises in the R&D and production of industrial robotics and integrated circuits. The Shenyang Machine Tool is a key national production base for CNC machine tools. Enterprises such as the Dalian Shipbuilding Industry and Dalian Machine Tool are state-invested and supported key enterprises in shipbuilding, marine engineering, and CNC machine tool manufacturing. When a city has geographical advantages or traditional industries have advantages, the state’s support for digitalization and key science and technologies in the high-end equipment manufacturing industry has contributed to the rapid development of the industry ahead of the construction of the three capacities of the city’s innovation chain. The offshore engineering equipment manufacturing industry has a relatively high driving force. It is a state-dominated industry and its core links are led by a few large state-owned businesses.

The above analysis shows that when a city has geographical advantages or traditional industry advantages, the state digital cultivation, key scientific and technological support for the high-end equipment manufacturing industry, located in the pillar industry of the city’s national economy, promotes the development of this industry faster than the three capacities for the construction speed of UICs. In summary, when all three capacities of the UIC are at a relatively high level and the development of each stage is balanced, the city’s innovation performance is at a medium level. Simultaneously, the level of development of SEIs in cities is influenced by the strength of state support for science and technology.

### Analysis of the characteristics of UICs with high innovation performance

**Fig 6:**
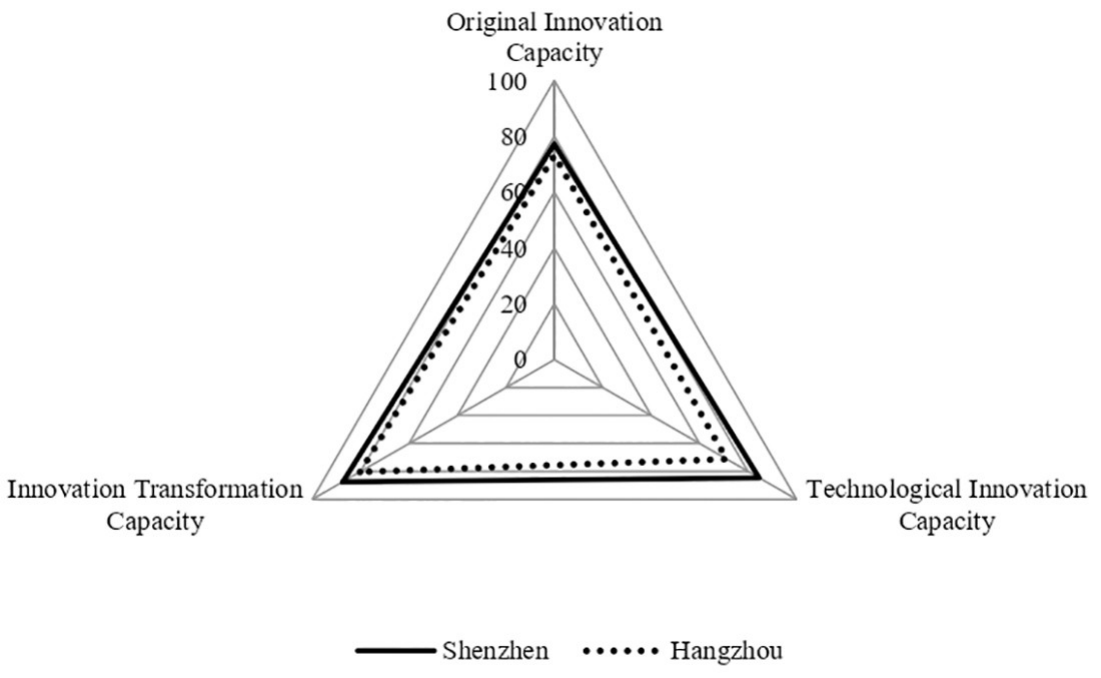
Three capacities of UICs in cities with high innovation performance.

Shenzhen and Hangzhou have relatively high innovation performances, as shown in Fig 6. Shenzhen’s original innovation capacity was 77.23, technological innovation capacity was 84.57, and innovation transformation capacity was 87.41, ranking 7th, 3rd, and 3rd in terms of the three capacities of the UIC among the 21 national innovation cities. Hangzhou’s original innovation capacity was 73.12, technological innovation capacity was 77.15, and innovation transformation capacity was 80.29, ranking 8th, 6th, and 5th. Both Shenzhen and Hangzhou enjoy a high level of the three capacities of the UIC, but the three capacities are uneven and innovation transformation capacity is higher than the original innovation and technological innovation capacities. In this case, the operational efficiency of the UIC is relatively high.

### Analysis of characteristics of UICs in cities with the highest innovation performance

**Fig 7:**
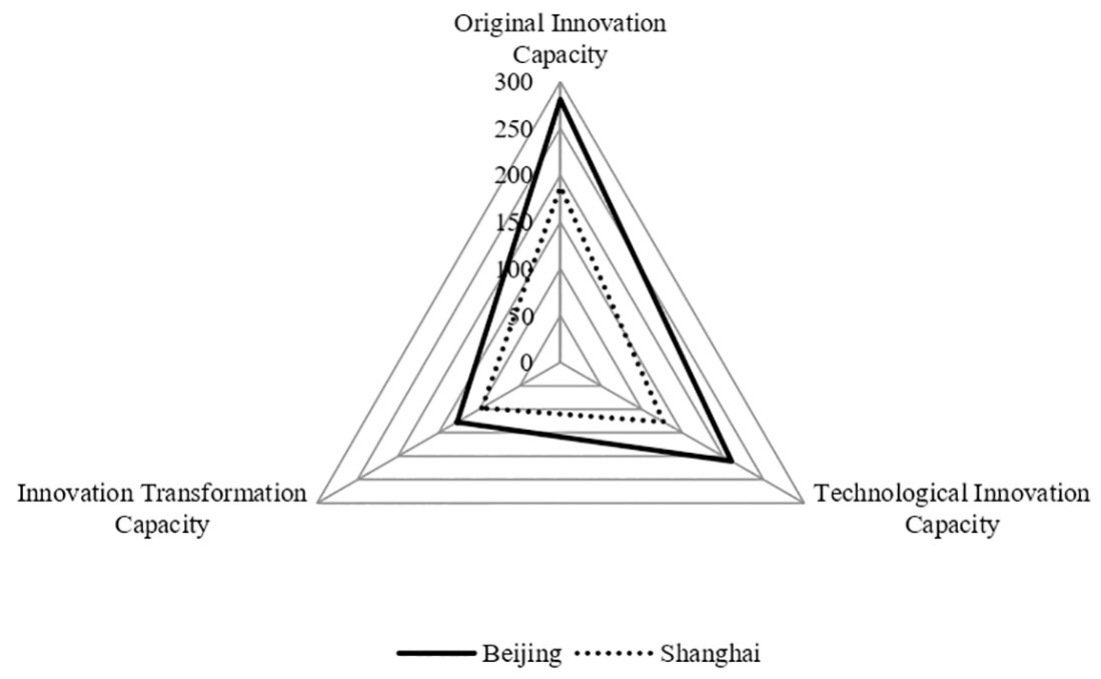
Three capacities of UICs in cities with the highest innovation performance.

Beijing and Shanghai had the highest innovation performance, as shown in Fig 7. Ranking 1st and 2nd respectively, the three capacities of the UIC in Beijing and Shanghai are both at the leading level in China. However, the three capacities differ greatly, with the original innovation capacity being the most prominent, and the original innovation capacity, technological innovation capacity, and innovation transformation capacity show a downward trend. When all three capacities are at their highest levels, the original innovation capacity drives technological innovation and the industrial application of new technologies, which in turn drives the rapid development of SEIs in the city.

### Hypothesis testing results

This study found that when the level of the three capacities of the UIC is generally low or the development of the three capacities is unbalanced, the city’s innovation performance is low; when the three capacities are at a high level and the original innovation capacity is the highest, the city’s innovation performance is at an intermediate level; and when the three capacities are at a high level and the innovation transformation capacity is the highest, the city’s innovation performance is relatively high. In summary, research hypotheses one and two are supported, namely, that the capacity of the UIC positively affects urban innovation performance, and the balanced development of the three capacities of the UIC positively affects urban innovation performance.

Urban innovation performance is highest when all three capacities of the UIC are at a high level and when the original innovation capacity is much higher than the technological innovation and innovation transformation capacity. Therefore, the third research hypothesis partially holds. In other words, there is no positive relationship between urban innovation performance and original innovation capacity. When urban innovation performance is at its highest level, original innovation capacity contributes significantly more to innovation performance than the other two capacities.

**Fig 8:**
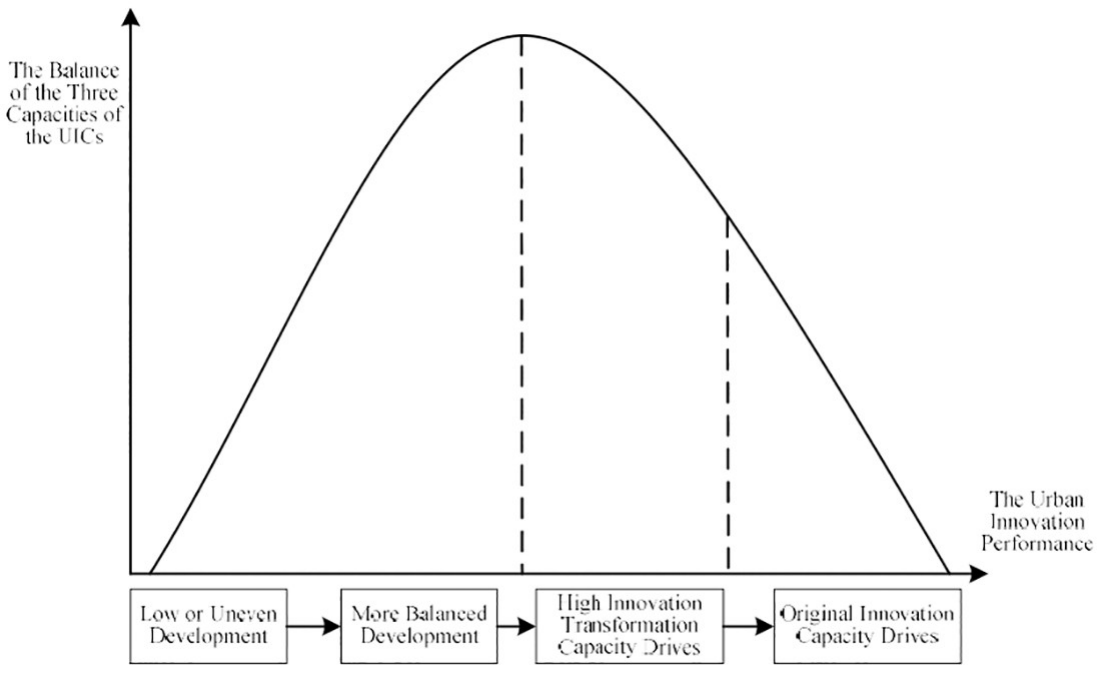
Relationship between the equilibrium degree of the three capabilities of the UIC and the innovation performance. Source: The authors.

An inverted U-curve illustrates the relationship between the balance of the three capacities of the UIC and the improvement in innovation performance. Specifically, when the three capacities of the UIC are low or uneven, urban innovation performance is relatively low. When the three capacities of the UIC are more balanced, the UIC operates with medium efficiency. At this moment, to continue to improve innovation performance, it is important to break the balance and focus on improving the innovation transformation capacity. When the innovation performance of a city is relatively high, the path to improving innovation performance is to vigorously develop its original innovation capacity. In summary, the improvement of the efficiency of the UIC goes through a process of “low or uneven development of the three capacities—more balanced development of the three capacities—the balance is broken and high innovation transformation capacity drives the improvement of the efficiency of the UIC—the imbalance is deepened, and high original innovation capacity drives the improvement of the efficiency of the UIC (Fig 8)”.

## Discussion

### Theoretical contribution

First, from the perspective of the process and stages of the innovation chain, the concept of UICs is analyzed and divided into three stages based on the characteristics of urban innovation activities: original, technological, and innovation transformation. This paper proposes that the construction of the UIC should be based on the construction of three capacities: original, technological, and innovation transformation capacity. An indicator system for evaluating the three capacities of UICs was established. The proposal of the UICs helps to conduct research on the relationship between the innovation capacity of each link of the innovation chain, the overall operation capacity of the innovation chain, and innovation performance from a new regional perspective. Innovation capacity is the ability to enhance existing products and technologies and create new products and technologies by applying knowledge to products and processes [94]. Knowledge creation and technological innovation are the bases of innovation ability [95]. Regional innovation capabilities not only focus on knowledge creation and technological innovation but also on transforming new technologies into new products to achieve regional economic growth, which is the commercial success of technological innovation. Owing to the increasing complexity and diversification of innovation activities, it is difficult for a single organization to independently complete the entire innovation process. As knowledge-intensive industries, SEIs are highly coupled with regional innovation systems [10]. During the innovation process, different businesses complete all innovation activities of knowledge creation, technological innovation, and, ultimately, large-scale market applications through the division of labor and cooperation [26]. The participants in the UCI complete the process of transforming the input of innovation elements into innovation output in the cross-regional division of labor and cooperation.

Second, this study finds that the capacity of the UIC positively affects urban innovation performance; the balanced development of the three capacities of the UIC positively affects urban innovation performance. When urban innovation performance is at its highest level, the contribution of original innovation capacity to innovation performance is significantly greater than the other two capacities. Innovation development between regions is not independent [96], and regional innovation activities are spatially correlated. However, geographical proximity is not a sufficient condition for regional innovation co-operation. The cyclic cumulative effect of market potential and its adsorption effect on adjacent areas promotes the aggregation of regional innovation elements [97]. Innovation is the result of the operation of complex systems. The UIC is a cyclic structural system with a feedback mechanism. The feedback mechanism improves the operational efficiency of the UIC, makes product innovation efficiency and knowledge innovation efficiency have obvious value chain spillover effects [89, 98], and promotes the improvement of urban innovation performance. With the improvement in the industrial application capacity of urban scientific and technological achievements, regionally advantageous industries have gradually grown and upgraded to SEIs [99, 100]. The expansion of business scale also promotes a corresponding improvement in its technological innovation resource level and technological innovation capacity. They have become important carriers for cities to carry out applied research and experimental development and to explore the application of basic research results.

Third, it clarifies the relationship between the construction of the three capacities of the UIC and the efficiency of the innovation chain. An inverted U-curve illustrates the relationship between the improvement of urban innovation performance and the balance of the three capacities of the UIC, and the improvement of the efficiency of the UIC goes through a process of “low or uneven development of the three capacities—more balanced development of the three capacities—the balance is broken and high innovation transformation capacity drives the improvement of the efficiency of the UIC, which deepens the imbalance, and high original innovation capacity drives the improvement of the efficiency of the UIC.” The three capabilities of the UIC promote the breakthrough of new technologies and their industrialization through innovative elements, such as human resources, capital, and platforms, to realize the economic and social development of the city. It helps cities optimize and adjust their economic structures, develop SEIs, and form a strong radiation and leading force on other regions [101]. However, cities have different factor endowments and innovation heterogeneity in their development. This leads to significant differences in innovation performance among different national innovative cities in China, which presents obvious urban hierarchical and regional heterogeneity [102].

### Practical implications

The three capacities of UIC in Guangzhou and Shenzhen were a relatively high. Guangzhou shows balanced development in the three capacities, with 80.94, 72.67, and 79.19, respectively, and Guangzhou’s innovation performance is at a medium level. In Shenzhen, the three capacities of the UIC scored 77.23, 84.57, and 87.41, respectively, and the innovation transformation capacity was higher than the original innovation and technological innovation capacities. Shenzhen’s innovation performance is relatively high. Although the three capacities of the UIC in Guangzhou and Shenzhen are both high, their innovation performances are different and they are at different stages of UIC development. Therefore, this study discusses strategies to improve the operational efficiency of the UIC in Guangzhou and Shenzhen by analyzing the construction of three chain capabilities.

Technology contract turnover is an important indicator for measuring the transformation of scientific and technological achievements, and the absorption of technology contract turnover reflects a city’s ability to industrialize scientific and technological achievements. Between 2016 and 2020, Guangzhou achieved remarkable results in terms of improving its innovation transformation capacity. The number of technology contract turnovers jumped to second place in Chinese cities. The number of national science and technology-based SMEs increased by 10,500 annually, ranking first in China. The number of listed companies in the science and technology sector moved to 7th in China. The number of high-tech enterprises in China was 12,174 and ranked 4th in China. After five years of development, although Guangzhou’s innovation transformation capacity has increased at a remarkable rate and ranks first in China, there is still a huge gap in terms of the number of listed companies in the science and technology sector, the number of national high-tech enterprises, and the number of global top 500 enterprises in comparison to cities such as Beijing and Shanghai. SEIs are leading industries that drive the rapid development of the regional economy only when they reach the stage of growth. The innovation transformation capacity directly reflects the efficiency of the UIC, industrialization ability and economic contribution of scientific and technological achievements in a city. When all three capacities of the UIC are at a high level, the path to improving the efficiency of the UIC is to focus on the innovation transformation capacity. Therefore, Guangzhou should improve the efficiency of its UIC by continuously increasing its innovation transformation capacity.

The number of patent applications is an important indicator of a city’s technological innovation. Shenzhen has been the at top among the large and medium-sized cities in terms of domestic patent applications, and its number of international patent applications has ranked first in China for 17 consecutive years. The absorption of technology contract turnover reflects the city’s ability to industrialize scientific and technological achievements, and Shenzhen’s technology contract turnover has ranked first among Chinese cities for 10 consecutive years in 2020. Shenzhen has strong innovation, transformation, and technological innovation capacity. Globally, the investment in basic research in technologically advanced cities with the core objective of building a global innovation center is generally 15%–25%of the total R&D investment. Shenzhen’s basic research expenditure from 2009 to 2015 accounted for 0.24%–0.92% of the total R&D expenditure. In 2016, Shenzhen began to increase its investment in basic research, and the proportion of basic research expenditure to R&D expenditure increased to 3.29% in 2020; however, it is still far lower than that of Beijing and Shanghai. Original innovation is the source of technological innovation, and the continuous improvement of technological innovation and innovation transformation capacities depends on the steady output of basic research. When the efficiency of the UIC has reached a relatively high level, the initiatives, breakthroughs, and driving forces of original innovation enhance the contribution of the original innovation capacity to urban innovation performance. At this stage, original innovation capacity boosts UIC efficiency. However, the investment cycle of basic research is long, with high risk and slow returns. Enterprises, society, capital, and markets lack incentives to invest in basic research. Therefore, at this stage, Shenzhen should optimize the layout of and increase investment in basic research by allowing the government to serve as the organizer of scientific and technological innovation and the main body of investment.

### Limitations and future research

First, the selection of urban innovation performance indicators was limited. Given the important role of SEIs in national economic development, this study uses the development level of SEIs to measure the innovation performance of cities. Since the statistical yearbook lacks data on the distribution, output value, and growth rate of SEIs in each city to reflect their development level, this study selected five industries in the fields of digital high-end equipment manufacturing and the digital economy, which play a key role in driving economic growth and have high industrial concentration as indicators to measure the innovation performance of cities. These data reflect the level of development SEIs to a certain extent. Therefore, to better build the capacity of the UIC and improve innovation performance, researchers should continue to explore indicators that can comprehensively reflect the innovation performance of cities, and further explore the correlation between the construction of the capacity of the UIC and innovation performance.

Second, owing to the limitations of the research methods, Type 1 and Type 2 error tests cannot be conducted when discussing the acceptance or rejection of a hypothesis, but the mediation effect test can better control for the probability of Type 1 and Type 2 errors [103]. In the future, when relevant statistical data becomes available, the acceptability of this study’s hypothesis should be further discussed through a mediation effect test.

Third, China had 293 prefecture-level cities. Recently, most cities have committed to constructing urban innovation capabilities to develop their economies. This study examines only some of the national innovative cities supported by the Chinese government and does not cover the entire country. In the future, all cities should be considered to explore UIC capacity development and innovation performance improvement strategies further.

## Supporting information

S1 File (ZIP)
