# Supplementary material for: Evaluation and construction of the capacities of urban innovation chains based on efficiency improvement
Source: PLoS One. 2022 Oct 26;17(10):e0274092. doi: 10.1371/journal.pone.0274092 (PMC9604944; doi:10.1371/journal.pone.0274092)
Supplement: S1 File — (ZIP) [file pone.0274092.s001.zip › data/Distribution of Strategic Emerging Industries in China National Innovative Cities.docx]

**Distribution of Strategic Emerging Industries in China National Innovative Cities**

| ***No.*** | ***City*** | ***Industries of Industrial Robotics Leading Manufacturers*** | ***Digital Economy Leading Manufacturers*** | ***Integrated Circuits Leading Manufacturers*** | ***Marine Engineering Equipment Leading Manufacturers*** | ***High-end CNC Machine Tools Leading Manufacturers*** | ***Environmental Protection Equipment Leading Manufacturers*** | ***The Number of Leading Manufacturers in Strategic Emerging Industries*** |
| --- | --- | --- | --- | --- | --- | --- | --- | --- |
| 1 | Beijing | -- | Tsinghua Unigroup Co., Ltd.,Beijing Bytedance Technology Co.,Ltd., Tencent Cloud Computing(Beijing) Company Limited, Beijing Baidu Netcom Science and Technology Co.,Ltd., Jingdong Digits Technology Holding Co.,Ltd., Beijing Express Hand Technology Co., Ltd., Beijing Megvii Technology Limited, Digital China(China)Ltd., Bank of Beijing Co.,Ltd. | Beijing Micro – electronics, Beijing Huachuang Integration Circuit Co.,Ltd., Beijing Zhongkexin Electronics Equipment Co.,Ltd. | -- | Beijing No.1 Machine Tool Plant, Beijing Machine Tool Institute Precision Mechatronics Co., Ltd. | Beijing Originwater Technology Co.,Ltd., Beijing Spc Environment Protection Tech Co.,Ltd., Beijing Sdl Technology Co.,Ltd., Beijing Greentec Acoustics Engineering Holding Co.,Ltd | 18 |
| 2 | Shanghai | Shanghai Step Electric Corporation | Pinduoduo (Shanghai) Network Technology Co., Ltd., Ant Blockchain Technology (Shanghai) Co., Ltd., Shanghai Hema Network Technology Co., Ltd., Shanghai SenseTime Intelligent Technology Co., Ltd. | Advanced Micro-Fabrication Equipment Inc. China, Shanghai Microelectronics Equipment Co., Ltd., Acm Research (Shanghai), Inc., Shanghai Ruili Information Technology Co., Ltd., Shanghai Kingstone Semiconductor Corp.,Shanghai Micson Industrial Automation Co., Ltd. | Shanghai Waigaoqiao Shipbuilding Co., Ltd., Shanghai Zhenhua Heavy Industries Co., Ltd., Shanghai Shipyard Co.,Ltd. | Shanghai Machine Tool Electrical Apparatus Factory Co.,Ltd | Shanghai Changshen Greening Engineering Co., Ltd. | 16 |
| 3 | Shenzhen | -- | Huawei Technologies Co., Ltd., ZTE Corporation, SZ DJI Technology Co., Ltd., Shenzhen TCL Lighting Technology Co.,Ltd, Byd Company Limited, Ping An Technology(Shenzhen)Co.,Ltd., ShenZhen Hanwuji Intelligent Technology Co.,Ltd., BGI Co-Win (Shenzhen) Equity Investment Fund Management Co., Ltd. | -- | China Merchants Heavey Ind.(szn) Co., Ltd. | -- | Dongjiang Environmental Company Limited | 10 |
| 4 | Hangzhou | -- | Hangzhou Hikvision DIGITAL Technology Co., Ltd., Aliyun Computing Co., Ltd., Hangzhou H3c Technologies Co., Ltd., Hangzhou Several Dreamworks Animation Technology Co., Ltd., Zhejiang Geely Holding (Group) Co., Ltd., Zhejiang Dahua Technology Co., Ltd. | -- | -- | -- | Focused Photonics (hangzhou), Inc. | 7 |
| 5 | Nanjing | Nanjing Panda Electronics Company Limited, Nanjing Estun Automation Co., Ltd. | -- | -- | -- | Nanjing No.2 Machine Tool Works, NanJing CNC Machine Tool Co., Ltd. | Cec Environmental Protection Co., Ltd. | 5 |
| 6 | Shenyang | Siasun ROBOT&AUTOMATION Co., Ltd. | -- | Kingsemi Co., Ltd., Piotech Inc., Shenyang Keyi Shixun Systems Engineering Co., Ltd. | -- | Shenyang Machine Tool (Group) Co., Ltd. | -- | 5 |
| 7 | Guangzhou | Guangzhou GSK Robot, Technology Co., Ltd. | Guangzhou Automobile Group Co., Ltd. | -- | -- | Guangzhou GSK Robot Technology Co., Ltd., Hans Laser | -- | 4 |
| 8 | Dalian | -- | -- | -- | Dalian Shipyard Group Co., Ltd., Cosco Shipyard Group Co.,Ltd. | Dalian Machine Tool（CNC）Co., Ltd., Kede Numerical Control Co.,Ltd | -- | 4 |
| 9 | Tianjin | -- | Didi Travel Technology Co., Ltd. | -- | -- | Tianjin No.1Machine Tool Co., Ltd., Tianjin Tianduan Press Co., Ltd. | -- | 3 |
| 10 | Chengdu | -- | -- | -- | -- | Chengdu Precise Cnc Machine Tool Co., Ltd., Chengdu Ningjiang Machine Tool Group Co., Ltd. | Chengdu Huanneng Demei Investment Limited | 3 |
| 11 | Qingdao | -- | Haier Smart Home Co., Ltd., Dawning International Information Industry Co., Ltd. | -- | Qingdao Beihai Shipping Co., Ltd. | -- | -- | 3 |
| 12 | Jinan | -- | Inspur Group Co., Ltd. | -- | -- | Jinan First Machine Tool Group Co., Ltd.,Jier Machine-Tool Group Co.,Ltd. | -- | 3 |
| 13 | Yangzhou | -- | -- | -- | -- | Yangzhou Metalforming Machine Tool Co., Ltd.,Yangli Group Corporation Ltd., Jiangsu Yawei Machine Tool Co., Ltd. | -- | 3 |
| 14 | Suzhou | -- | -- | -- | -- | Suzhou Electric Processing Machine Tool Research Institute Co., Ltd. | Create Technology and Science Co., Ltd., Kelin Environmental Protection Equipment,inc. | 3 |
| 15 | Chongqing | -- | -- | -- | -- | Chongqing Machine Tool (Group) Co., Ltd. | Chongqing Sanfeng Environment Group Corp., Ltd. | 2 |
| 16 | Wuhan | -- | -- | -- | -- | Wuhan Heavy Duty Machine Tool Group Corporation, Wuhan Huagong Laser Engineering Co., Ltd. | -- | 2 |
| 17 | Hefei | -- | Iflytek Co., Ltd. | -- | -- | -- | Anhui Guozhen Environment Protection Technology Joint Stock Co., Limited. | 2 |
| 18 | Kunming | -- | -- | -- | -- | Kunming Kunji Group Co., Yunnan CY Group Co., Ltd. | -- | 2 |
| 19 | Wuxi | -- | -- | -- | -- | -- | Wuxi Xuelang Environmental Technology Co., Ltd., Jiangsu Yihuan Group Co., Ltd. | 2 |
| 20 | Nantong | -- | -- | -- | China Merchants Heavy Industry (Jiangsu) Co., Ltd., Jiangsu Rongsheng Heavy Industry Co., Ltd. | -- | -- | 2 |
| 21 | Baoji | -- | -- | -- | -- | Baoji Machine Tool Group Co., Ltd., Qinchuan Machine Tool & Tool Group Share Co., Ltd. | -- | 2 |
| 22 | Changsha | -- | -- | -- | -- | -- | Yonker Environmental Protection Co., Ltd. | 1 |
| 23 | Changzhou | -- | -- | -- | -- | -- | WELLE Environmental Group Co., Ltd. | 1 |
| 24 | Jiaxing | -- | -- | -- | -- | -- | Jiehua Holdings Co., Ltd. | 1 |
| 25 | Wuhu | Efort Intelligent Equipment Co., Ltd. | -- | -- | -- | -- | -- | 1 |
| 26 | Foshan | -- | Midea Group Co., Ltd. | -- | -- | -- | -- | 1 |
| 27 | Fuzhou | -- | -- | -- | -- | -- | Fuzhou Longma Environmental Sanitation Engineering Co., Ltd. | 1 |
| 28 | Yantai | -- | -- | -- | Yantai CIMC Offshore Co., Ltd. | -- | -- | 1 |
| 29 | Harbin | Harbin Boshi Automation Co., Ltd. | -- | -- | -- | -- | -- | 1 |
| 30 | Taizhou | -- | -- | -- | Yangzijiang Shipbuilding group | -- | -- | 1 |
| 31 | Shaoxing | -- | -- | -- | -- | -- | Zhejiang Feida Environmental Science & Technology Co.,Ltd. | 1 |
| 32 | Hanzhong | -- | -- | -- | -- | Shaanxi Hanjiang Machine Tool Co.,Ltd. | -- | 1 |
| 33 | Xining | -- | -- | -- | -- | Qinghai Huading Industrial Co.,Ltd. | -- | 1 |
| 34 | Yichang | -- | -- | -- | -- | -- | Tus Environmental Science And Technology Development Co.,Ltd. | 1 |
| 35 | Longyan | -- | -- | -- | -- | -- | Fujian Longking Co.,Ltd. | 1 |

Source：Industrial Innovation and Competition Map (2018)
