# Supplementary material for: Evaluation and construction of the capacities of urban innovation chains based on efficiency improvement
Source: PLoS One. 2022 Oct 26;17(10):e0274092. doi: 10.1371/journal.pone.0274092 (PMC9604944; doi:10.1371/journal.pone.0274092)
Supplement: S1 File — (ZIP) [file pone.0274092.s001.zip › data/Three Capacities of the Urban Innovation Chain of China National Innovative Cities.docx]

**Three Capacities of the Urban Innovation Chain of China National Innovative Cities**

| ***NO.*** | ***City*** | ***Original Innovation Capacity*** | ***Technological Innovation Capacity*** | ***Innovation Transformation Capacity*** |
| --- | --- | --- | --- | --- |
| 1 | Beijing | 280.7 | 210.42 | 127.39 |
| 2 | Shanghai | 187.72 | 126.62 | 97.06 |
| 3 | Shenzhen | 77.23 | 84.57 | 87.41 |
| 4 | Hangzhou | 73.12 | 71.15 | 80.29 |
| 5 | Nanjing | 83.43 | 72.68 | 74.73 |
| 6 | Shenyang | 67.49 | 59.15 | 64.7 |
| 7 | Guangzhou | 80.94 | 72.67 | 79.19 |
| 8 | Dalian | 65.21 | 62.63 | 48.45 |
| 9 | Tianjin | 72.17 | 63.64 | 51.08 |
| 10 | Chengdu | 71 | 66.1 | 71.58 |
| 11 | Qingdao | 71 | 65.47 | 68.12 |
| 12 | Jinan | 66.18 | 69.16 | 64.67 |
| 13 | Yangzhou | 42.51 | 56.1 | 46.28 |
| 14 | Suzhou | 54.15 | 68.72 | 82.25 |
| 15 | Chongqing | 90.21 | 18.52 | 36.75 |
| 16 | Wuhan | 77.52 | 70.39 | 74.93 |
| 17 | Hefei | 70.66 | 68.11 | 57.75 |
| 18 | Kunming | 61.8 | 56.48 | 50.38 |
| 19 | Wuxi | 59.44 | 65.57 | 67.01 |
| 20 | Nantong | 42.31 | 55.11 | 54.71 |
| 21 | Baoji | 25.47 | 39.8 | 21.89 |
| 22 | Changsha | 71.46 | 68.94 | 60.42 |
| 23 | Changzhou | 47.11 | 63.98 | 63.32 |
| 24 | Jiaxing | 43.2 | 45.08 | 50.67 |
| 25 | Wuhu | 40.42 | 64.05 | 39.48 |
| 26 | Foshan | 30.73 | 43.59 | 54.04 |
| 27 | Fuzhou | 61.31 | 50.19 | 48.1 |
| 28 | Yantai | 48.91 | 52.59 | 55.67 |
| 29 | Harbin | 65.37 | 55.88 | 60.47 |
| 30 | Taizhou | 36.01 | 46.54 | 46.45 |
| 31 | Shaoxing | 27.04 | 52.51 | 28.59 |
| 32 | Hanzhong | 11.22 | 9.47 | 17.79 |
| 33 | Xining | 41.62 | 24.96 | 30.54 |
| 34 | Yichang | 18.64 | 45.49 | 35.19 |
| 35 | Longyan | 25.16 | 19.48 | 17.64 |

Source: The 2020 Report on the Innovation Capacity of National Innovative Cities (2020).
